# Effects of an indole derivative on cell proliferation, transfection, and alternative splicing in production of lentiviral vectors by transient co-transfection

**DOI:** 10.1371/journal.pone.0297817

**Published:** 2024-06-04

**Authors:** Nataly Carolina Mier, Donald Keith Roper

**Affiliations:** Department of Biological Engineering, Utah State University, Logan, Utah, United States of America; University of South Florida, UNITED STATES

## Abstract

Lentiviral vectors derived from human immunodeficiency virus type I are widely used to deliver functional gene copies to mammalian cells for research and gene therapies. Post-transcriptional splicing of lentiviral vector transgene in transduced host and transfected producer cells presents barriers to widespread application of lentiviral vector-based therapies. The present study examined effects of indole derivative compound IDC16 on splicing of lentiviral vector transcripts in producer cells and corresponding yield of infectious lentiviral vectors. Indole IDC16 was shown previously to modify alternative splicing in human immunodeficiency virus type I. Human embryonic kidney 293T cells were transiently transfected by 3^rd^ generation backbone and packaging plasmids using polyethyleneimine. Reverse transcription-quantitative polymerase chain reaction of the fraction of unspliced genomes in human embryonic kidney 293T cells increased up to 31% upon the indole’s treatment at 2.5 uM. Corresponding yield of infectious lentiviral vectors decreased up to 4.5-fold in a cell transduction assay. Adjusting timing and duration of IDC16 treatment indicated that the indole’s disruption of early stages of transfection and cell cycle had a greater effect on exponential time course of lentiviral vector production than its reduction of post-transcriptional splicing. Decrease in transfected human embryonic kidney 293T proliferation by IDC16 became significant at 10 uM. These findings indicated contributions by early-stage transfection, cell proliferation, and post-transcriptional splicing in transient transfection of human embryonic kidney 293T cells for lentiviral vector production.

## Introduction

Lentiviral vectors (LVs) are one of the two most widely applied viral vectors in gene therapy [1]. They efficiently deliver genes to dividing and non-dividing cells, providing long-term transgene expression, a large cloning capacity of up to 9 kb, low immunogenicity, and broad tropisms [2–4]. For these reasons they are widely used in research (e.g., animal models through CRISPR/CAS [5, 6]) and clinical (e.g., gene therapies [7]) settings. As of 2020, LVs have been utilized in over 200 clinical trials [8] to treat a range of diseases [4, 9] from Parkinson’s disease [10] to cancer [11–14]. For current gene therapies, LV yield is a limiting factor [15]. The production of LVs ranges from ~ $300,000 to $500,000 [16, 17] which represents 48% of the cost of LV-based therapies [8]. The widespread utility of LVs has motivated improvements to their safety, efficacy, and manufacture.

Most LVs are derived from human immunodeficiency virus type I (HIV-1). To minimize risk of generating replication-competent LVs, 3^rd^-generation LV is produced via transient transfection of primarily human embryonic kidney cells (HEK) 293 [18–27] by an engineered backbone plasmid bearing the transgene plus three helper plasmids [22] that comprise a minimal set of constitutively expressed genes. Host HEK 293T cells enable productive LV preparation due to expression of SV40 T-antigen [28, 29], susceptibility to virus budding from the membrane [30, 31], and adaptability to suspension culture [20, 32, 33]. Host cells transduced by LVs, however, potentiate genotoxicity due to aberrant splicing [34]. Moiani *et al*. [35] and Cesana *et al*. [36] identified chimeric LV-cellular transcripts attributable to transgene splice sites and characterized vector-induced aberrant splicing to decrease its potential for genotoxicity. Knight *et al*. [37] modified splice sites in an LV construct to minimize post-integration aberrant splicing events in host cells. Sertkaya *et al*. [38] found that ~95% of 3rd-generation backbone transcripts were spliced in adherent producer cells and showed deletions in *env* gene increased unspliced transcripts to 15% of the total, yet LV production remained unaffected. Unlike HIV-1 replication in which splicing is requisite [39–41], 3^rd^ generation plasmids do not require splicing to generate LV [24]. Sertkaya *et al*. [38] indicated that further reduction in unintended splicing may increase titer of functional LV.

Alteration of HIV-1 splicing [42] by small organic molecules has been examined for possible antiviral treatments [43, 44]. A large-scale screening of indole derivative compounds (IDCs) found that indole derivative IDC16 inhibited spliced HIV-1 transcripts, thereby reducing levels of key viral proteins in infected cells [45, 46]. Bakkour *et al*. measured inhibition of HIV-1 replication via IDC16-disrupted splicing using ELISA of capsid protein p24 [45] as a surrogate for functional virus [47]. A 47% increase in unspliced viral transcripts following 0.5 uM IDC16 treatment was attributed to inhibition by IDC16 of the RNA-binding protein SF2/ASF involved in splicing [45]. Janus Kinase (JAK) inhibitor [48, 49] Filgotinib, used to treat autoimmune diseases [50], was also reported to inhibit HIV-1 transcription with a concomitant change in the relative copies of spliced and unspliced HIV-1 RNA. A mechanism for the inhibition, however, was not elucidated [51].

The present study examined effects of indole derivative IDC16 and Filgotinib on splicing of LV transcripts in adherent human embryonic kidney (HEK) 293T producer cells. Corresponding yields of transduction-competent LV were evaluated by cell transduction assay. Evaluating the time-course of LV production and modifying the timing and duration of treatment indicated that disruption by the small molecule of early stages of transfection related to plasmid trafficking and cell cycle had a larger effect on LV yield than subsequent post-transcriptional splicing. These results expand understanding of post-transcriptional alternative splicing in LV production by transient co-transfection in HEK293T

## Materials and methods

### Cell culture

HEK293T/17 adherent cells (ATCC CRL-11268) were cultured in a supplemented Dulbecco’s Modified Eagle Medium (DMEM; Gibco 11960044), referred to here as SDMEM, containing a final concentration of 1% 100 X GlutaMAX-I (Gibco 35050061), 1% 100 X MEM non-essential amino acids (NEAA; Gibco 11140050), 1 mM sodium pyruvate (Gibco 11360070) and 10% fortified bovine calf serum (HyClone SH30087.03) [52]. No antibiotic was added to the culture media. The culture was maintained at 37°C in a humidified atmosphere with 5% CO_2_.

### Vector production

HEK293T/17 cells were seeded in SDMEM in 96-well plates at a density of 7.5x10^3^ or 8x10^3^ cells per well 72 h prior to transfection. The cells were transiently co-transfected with 3^rd^ generation LV-MAX lentiviral packaging mix (Gibco A43237) and pLJM1-EGFP (Addgene Plasmid #19319) as backbone vector using 1:3 DNA/polyethyleneimine (PEI) ratio, 3:2 packaging/backbone plasmids ratio, and a total of 55 ng of DNA per well, which corresponded to 0.57 ug / 10^6^ cells. The DNA and PEI were complexed in Opti-MEM (Gibco 31985070) for 15 min. Media with PEI-DNA complexes was removed 6 hours post-transfection. After a 1X Dulbecco’s phosphate-buffered saline (DPBS; Gibco 14190144) wash, transfection enhancer medium consisting of Opti-MEM, 5% KnockOut Serum Replacement (KSR; Gibco 10828010), and 1 mM sodium butyrate (Na Bu) was added to the cells. Viral supernatant was collected 24 h post-transfection and centrifuged at 2000 x g for 30 or 45 s. Adherent cells were processed for ‘Cell RNA isolation’ as detailed below.

### Small molecules treatments in vector production

Tetracyclic IDC16 indole derivative and Filgotinib were tested as alternative splicing modifiers in LV production by adherent HEK293T/17 cells. IDC16 was obtained from the chemical library of Institut Curie–Centre National de la Recherche Scientifique and Filgotinib from Selleckchem (GLPG0634). Each compound was dissolved at 1 mM in dimethylsulfoxide (DMSO). Subsequent dilutions of these stock solutions of IDC16 and Filgotinib, or DMSO control, were added to transfection mixture as well as to the enhancer medium. Different final concentrations per well of the small molecules and DMSO were tested. IDC16: 0.5, 1, 2.5, and 10 uM, Filgotinib: 2, 4, 6, and 10 uM, and DMSO: 7, 14, 35, 56 and 140 mM. Three to six replicates of each treatment and controls were performed.

Experiments to differentiate effects of IDC16 from 0 to 6 and 6 to 24 h post-transfection, respectively, were performed as described above with certain modifications. At 6 hours post-transfection, treatment was removed from cells (none, small molecule diluted in DMSO, and DMSO) and only transfection enhancer solution was added into the wells conserving the original concentration per well. Viral supernatant was collected at 10-, 16-, and 24-hours post-transfection from independent 96-well plates for transduction. In another experiment, the treatment was added only at the time of the enhancer addition (6 hours post-transfection). In these studies, samples were also collected 24 hours post-transfection.

### Transfection efficiency

Transfection efficiency was determined by expression of Enhanced Green Fluorescent Protein (EGFP) in transfected cells at 6 h post- transfection. Fluorescent images of 2.3 mm^2^ area per well per replicate were captured employing Cytation 1 Cell Imaging Multi-Mode Reader (BioTek CYT1FAV) with 40X magnification and EX 485/20 nm, EM 528/20 nm filter. BioTek Gen5 software v3.10 was employed to analyze images and estimate fluorescent cell counts.

### Cell proliferation and morphology

Effects of indole on cell proliferation and morphology were evaluated by label-free cell count and brightfield microscopy at 24 h post-transfection. This method was preferable to e.g., MTT assay due to fewer limitations to assay validation [53–61] such as interferences from potentially confounding factors, e.g., cell culture variations, DMSO, optical effects, cell manipulation, and transfection effects on metabolic activity [62–73]. Cells were imaged at 40X magnification using the high contrast brightfield detection channel of the Cytation 1 Cell Imaging Multi-Mode Reader (BioTek CYT1FAV). BioTek Gen5 software v3.10 was used to estimate cell counts per 2.3 mm^2^ area per well per replicate.

### Supernatant RNA isolation

RNA from the viral supernatant of adherent cells was extracted following a modified version of a protocol by Ranheim *et al*. [74]. Briefly, the centrifuged viral supernatant was treated with 4.5% sterile-filtered Triton X solution for a final concentration of 0.9% detergent per vial. The mixture was stored at –80°C until analysis by qRT-PCR. On the day of analysis, the samples were thawed at room temperature, vortexed for 10 s, centrifuged at 2000 x g for 1 min, and diluted 1:40 in 10 mM Tris pH 7.0.

### Cell RNA isolation

HEK293T/17 transfected cells were washed with 1X Dulbecco’s phosphate-buffered saline (DPBS; Gibco 14190144) after supernatant collection at 24 h post-transfection. Cells were triturated from the bottom of the well by pipetting 100 uL of Opti-MEM up and down. The extract was transferred to a sterile polymerase chain reaction (PCR) tube and stored at– 80°C for 24 h. RNA from cell extract was isolated using a lysis buffer with a final concentration per sample of 4 M NaCl, 1 mM ethylenediaminetetraacetic acid (EDTA), and 10 mM Tris pH 7.2. The samples were thermally treated at 70°C for 15 minutes in a water bath. The lysate was centrifuged at 5000 x g for 5 min at 4°C. 100% ethanol (EtOH) was added dropwise to the cell lysate to achieve a 35% v/v concentration of ethanol. The mixture was transferred to a spin column attached to a vacuum manifold. The silica column was previously treated with a buffer solution of 2 M NaCl, 5 mM Tris pH 7.2, and 0.5 mM EDTA. Two washing steps were performed with wash buffer 1 (1.5 M NaCl, 20% EtOH, 10 mM Tris pH 7.2) and wash buffer 2 (0.1 M NaCl, 80% EtOH, 10 mM Tris pH 7.2). The nucleic acid was eluted twice from the column with 50 and 100 uL of pre-warmed 10 mM Tris pH 7.0. Each dilution consisted of a centrifugation step of 500 x g for 1 min at 25°C, a heating step at 71°C for 1 min, and another centrifugation step of 10 000 x g for 2 min at 25°C. The nucleic acid was stored at -20°C until use.

### Quantitative RT-PCR

Reverse transcription quantitative real-time PCR (RT-qPCR) was performed to quantitate nucleic acid extracted from transfected cells and viral supernatant from LV production in adherent cells using qScript One-Step Fast SYBR Green RT-qPCR Kit Low ROX (Quantabio 95089–200) and QuantiStudio 3 System (Thermofisher). The reaction was carried out in a 10 uL reaction mixture volume with a primer concentration of 0.2 uM, 4 uL of nucleic acid, 1X master mix, and 1X reverse transcriptase. The employed primers were 5’-TGTGTGCCCGTCTGTTGTGT-3’/5’-GAGTCCTGCGTCGAGAGAGC-3’ [75] (forward/reverse), 5’-TCTCGACGCAGG ACTCG-3’/5-‘TACTGACGCTCTCGCACC-3’ [38], and primer/probe set for RNAseP (IBFQ) reference gene (IDT 10007009). The first set of primers spans the U5-Psi sequence of the lentiviral vector and detects spliced and unspliced (total) LV genomic RNA. The second set spans the splicing donor 1 (SD1) sequence of the lentiviral vector and detects only unspliced viral genomic RNA. Absolute quantification of total, unspliced LV genomic RNA, and reference gene were determined using a standard curve of the pLJM1-EGFP DNA plasmid (Addgene Plasmid #19319) and Hs_RPP30 Positive Control DNA plasmid (IDT 10006626). Thermal cycling conditions consisted of cDNA synthesis at 50°C for 10 min, Taq DNA polymerase activation at 95°C for 5 min, and 40 cycles of the two-stage-temperature profile of 10 s at 95°C and 30 s at 60°C.

### Quantification of functional viral vector

HEK293T/17 cells were plated in SDMEM in 96-well plates at a density of 3.5 x10^3^ or 4 x10^3^ cells per well 96 h prior to transduction. Media was replaced 24 h before transduction. On the day of transduction, 60% of the media was extracted from the wells and 0.3 uL of centrifuged viral supernatant was dispensed in the remaining SDMEM. The 96-well plate was wrapped in parafilm and centrifuged at 100 g for 10 min. The previously removed media was reconstituted with fresh SDMEM 2 h post-transduction. The media volume was doubled 24 h after inoculation. The cells were incubated for 48 h and imaged using Cytation 1 Cell Imaging Multi-Mode Reader (BioTek CYT1FAV). Fluorescent microscopy is reported as a quantitative alternative method to flow cytometry [52, 76–80], provides comparable results [81], supports our economic, high-throughput, 96-well plate, adherent-culture workflow, and resulted in values within ranges reported for production of LV [82–84] (see Fig 2A).

Fluorescent images of the whole well were captured with 40X magnification and EX 485/20 nm, EM 528/20 nm filter. BioTek Gen5 software v3.10 was employed to analyze images and estimate fluorescent cell counts. Automatic cell counts were corrected manually. The functional transducing units (TUs) were calculated as:

$$\frac{TU}{ul}=\left(\frac{F}{V}\right)$$ (1)

F = number of fluorescent cells

V = volume of virus supernatant added to inoculate each well

For studies focused on differential effects of IDC16, 3, 1, and 0.75 uL of centrifuged viral supernatant were used to transduce cells at 10, 16, and 24 h, respectively.

### Statistics

**Fig 1:**
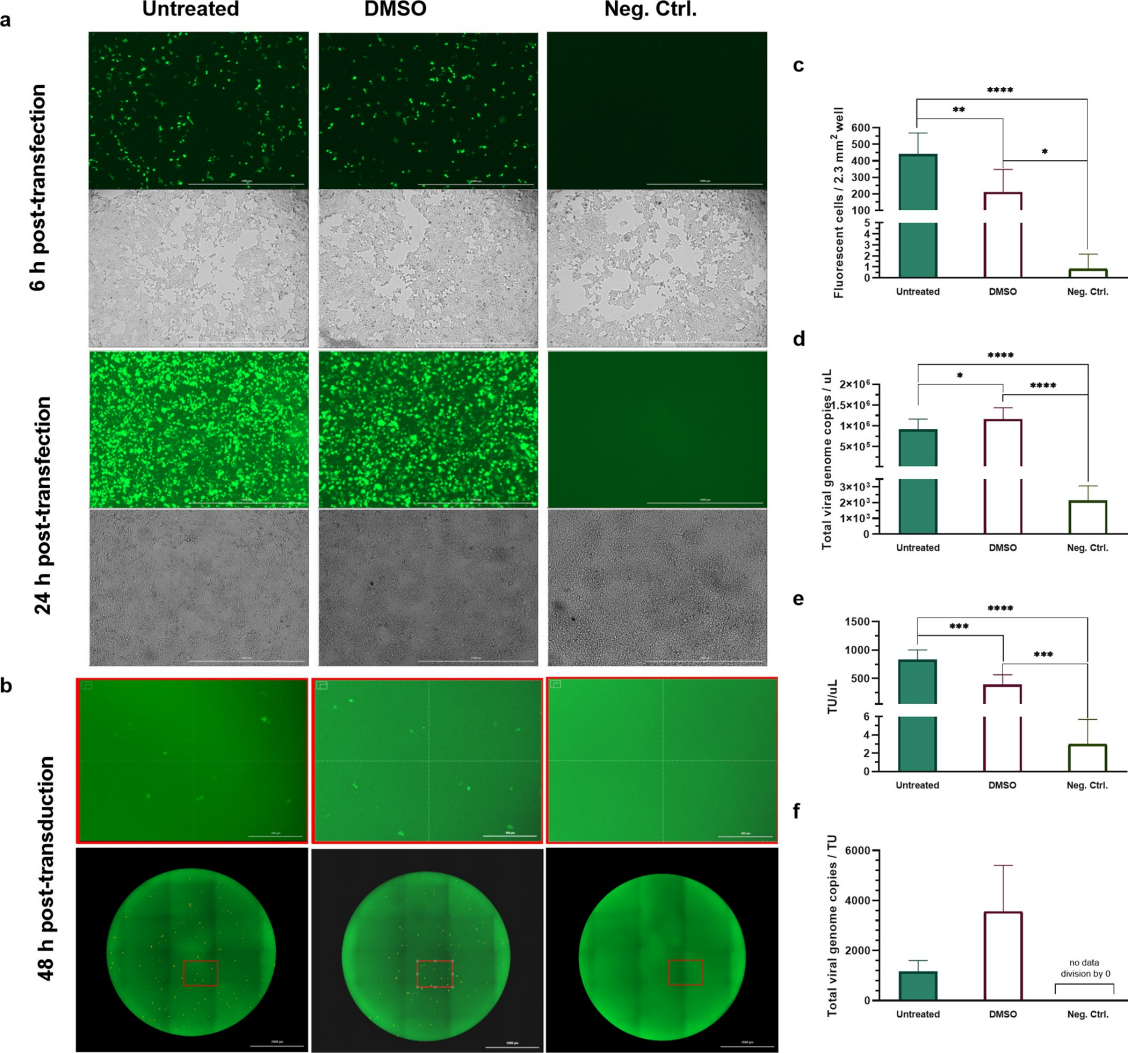
Green fluorescent protein (GFP) expression identified transfected cells in (a) and transduced cells in (b). (a) GFP expression and cell confluency in a well section at 6 and 24 h post-transfection. (b) GFP expression by cells transduced via supernatant from transfected cells without or with DMSO imaged 24 h post-transfection in a well (below) and section (above). Untreated and DMSO-treated transfected cells expressed GFP; non-transfected cells did not. (c) GFP expression per 2.33 mm^2^ of well measured at 6 h post-transfection (n = 6). (d) Total genome RNA viral copies per uL of supernatant 24 h post-transfection (n = 5 to 6). (e) Transduction units (TUs) or infectious virus per uL of supernatant (n = 6). (f) Ratio of total viral genomes to TU (n = 5 to 6). Bars and error bars show mean and SD. * indicates significant differences at p ≤ 0.05, ** at p ≤ 0.01, *** at p ≤ 0.001, **** at p ≤ 0.0001, and ns are ‘not significant’ differences.

**Fig 2:**
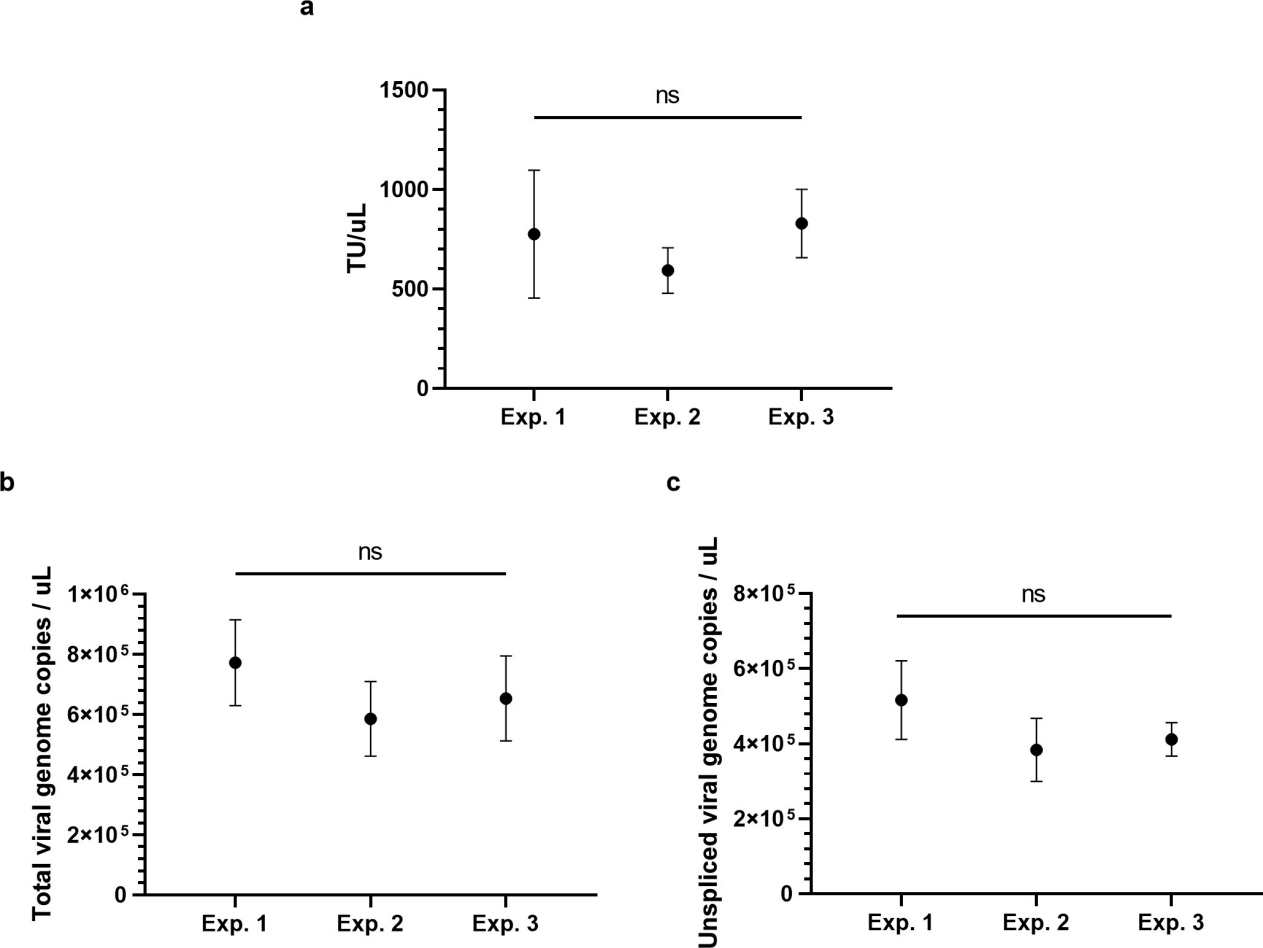
Infectious virus and viral genome copies remained consistent across three untreated cell transfections. (a) Cell transduction assay of TUs (n = 4 to 6), (b) total viral genome copies in cell extract (n = 4 to 5), and (c) unspliced viral genome copies in cell extract (n = 4 to 5). Symbols and error bars show mean and SD. ns are ‘not significant’ differences.

The normality and homogeneity of variance of the data were tested with Q-Q plot and Shapiro Wilk test and Brown-Forsythe test, respectively. Variables violating assumptions were transformed to reciprocal or sin. Differences between groups were evaluated by one-way ANOVA with Tuckey post-hoc test considering a p-value of < 0.05. Data is presented in means with error bars showing standard deviation (SD). N of each group is indicated in figure legends. Sample size was selected in order to enable significance of the results. Outliers were identified by Tukey’s Hinges test and excluded from analysis in Figs 1D, 2B, and 2C. Differences in the statistical analysis due to exclusion of outliers are detailed in S1 File. P values were identified in the figures by asterisks: * p ≤ 0.05, ** p ≤ 0.01, *** p ≤ 0.001, **** p ≤ 0.0001. Tukey’s Hinges test was performed in SPSS 25.0 (IBM, Armonk, NY, USA). All other statistical analyses were performed in GraphPad v8 (Prism, Irvine, CA, USA). See S1 File for more detailed information on the statistical analysis.

**Fig 3:**
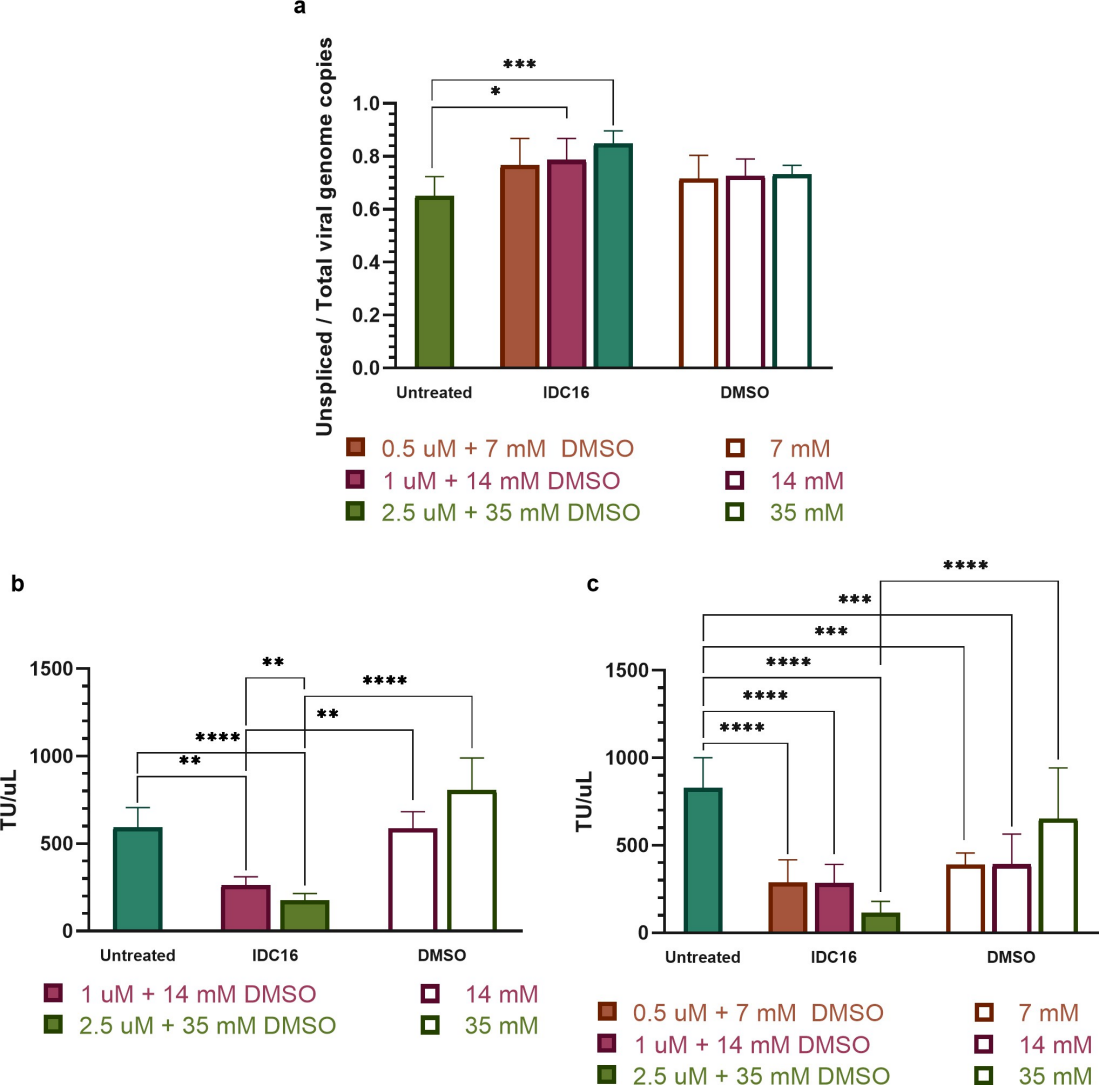
IDC16 increased the proportion of unspliced viral genomes in cells and reduced infectious LV production. (a) Ratio of unspliced to total genome RNA viral copies (n = 6). (b) and (c) Infectious LV measured by cell transduction assay (TU/uL) for IDC16 treatment from 1 to 2.5 uM (n = 4) and from 0.5 to 2.5 uM (n = 6), respectively. Bars and error bars show mean and SD. * indicates significant differences at p ≤ 0.05, ** at p ≤ 0.01, *** at p ≤ 0.001, and **** at p ≤ 0.0001.

**Fig 4:**
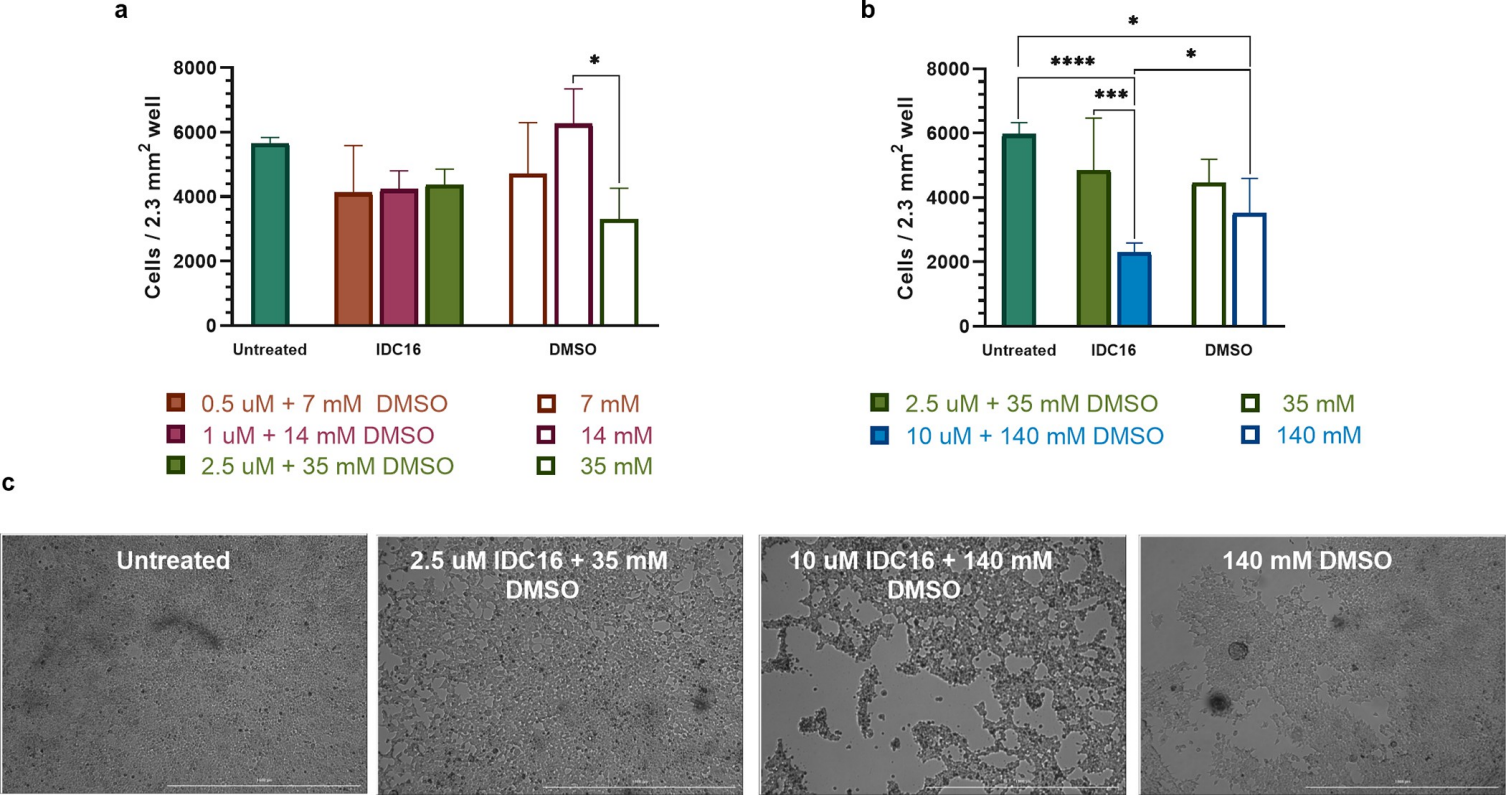
IDC16 was detrimental to cell proliferation and morphology in transfected cells. (a) Cell proliferation at 0.5–2.5 uM IDC16 (n = 3). b) Cell proliferation at 2.5–10 uM IDC16 (n = 4). (c) Images at 24 h post-transfection for untreated cells, IDC16 treated cells, and DMSO-only treated controls at different dilutions. Bars and error bars show mean and SD. * indicates significant differences at p ≤ 0.05, ** at p ≤ 0.01, and *** at p ≤ 0.001.

**Fig 5:**
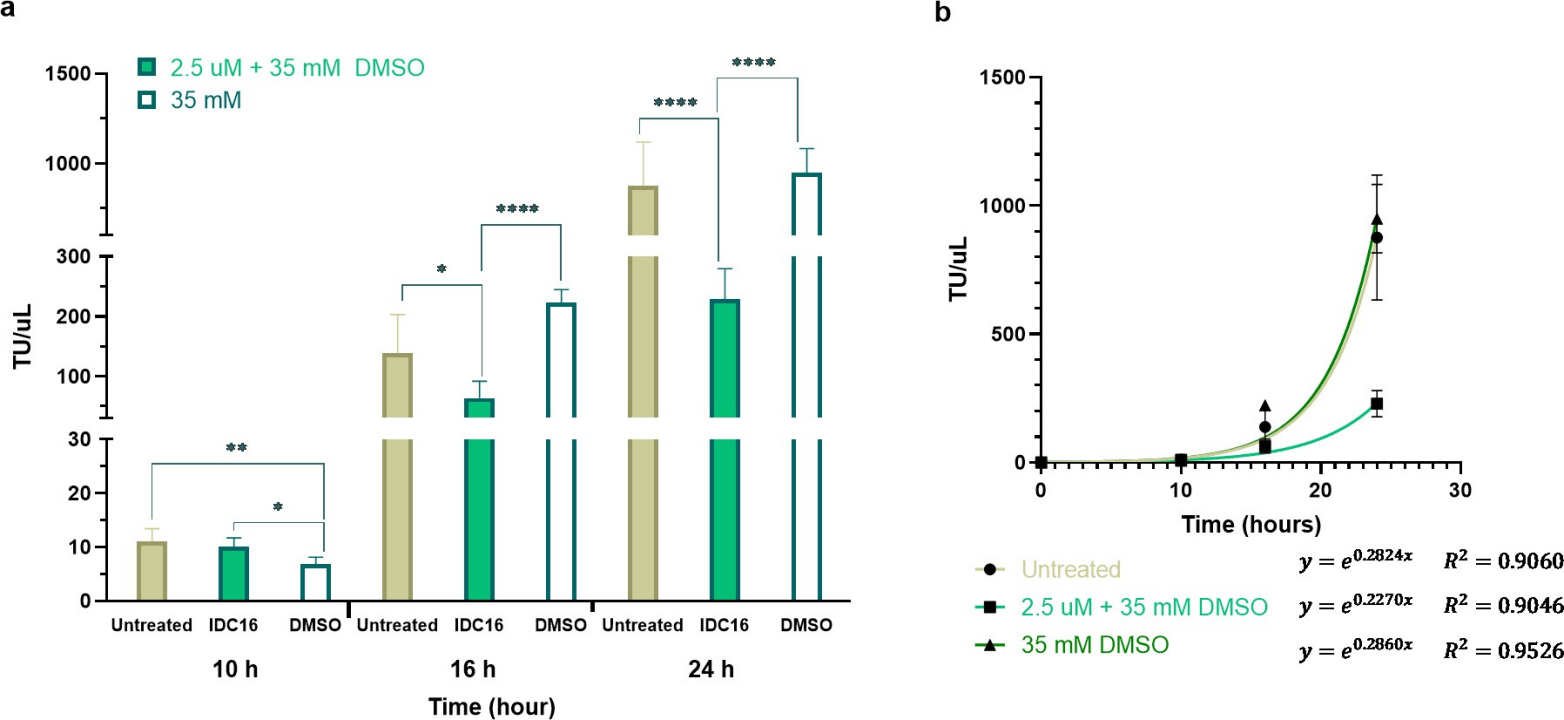
Time trajectories of LV production post-transfection. (a) Cell transduction assay and (b) production trajectory of LV in TU/uL at 10, 16, and 24 h post-transfection for untreated cells, 2.5 uM IDC16 + 35 mM DMSO treatment, and 35 mM DMSO control (n = 5 to 6). At 6 h post-transfection, treatments (none, IDC16 + DMSO or DMSO) were removed and washed out with DPBS from the transfected cell and only the enhancer solution (NaBu + KSR) was added. Points and error bars show mean and SD. * indicates significant differences at p ≤ 0.05, ** at p ≤ 0.01, and **** at p ≤ 0.0001.

**Fig 6:**
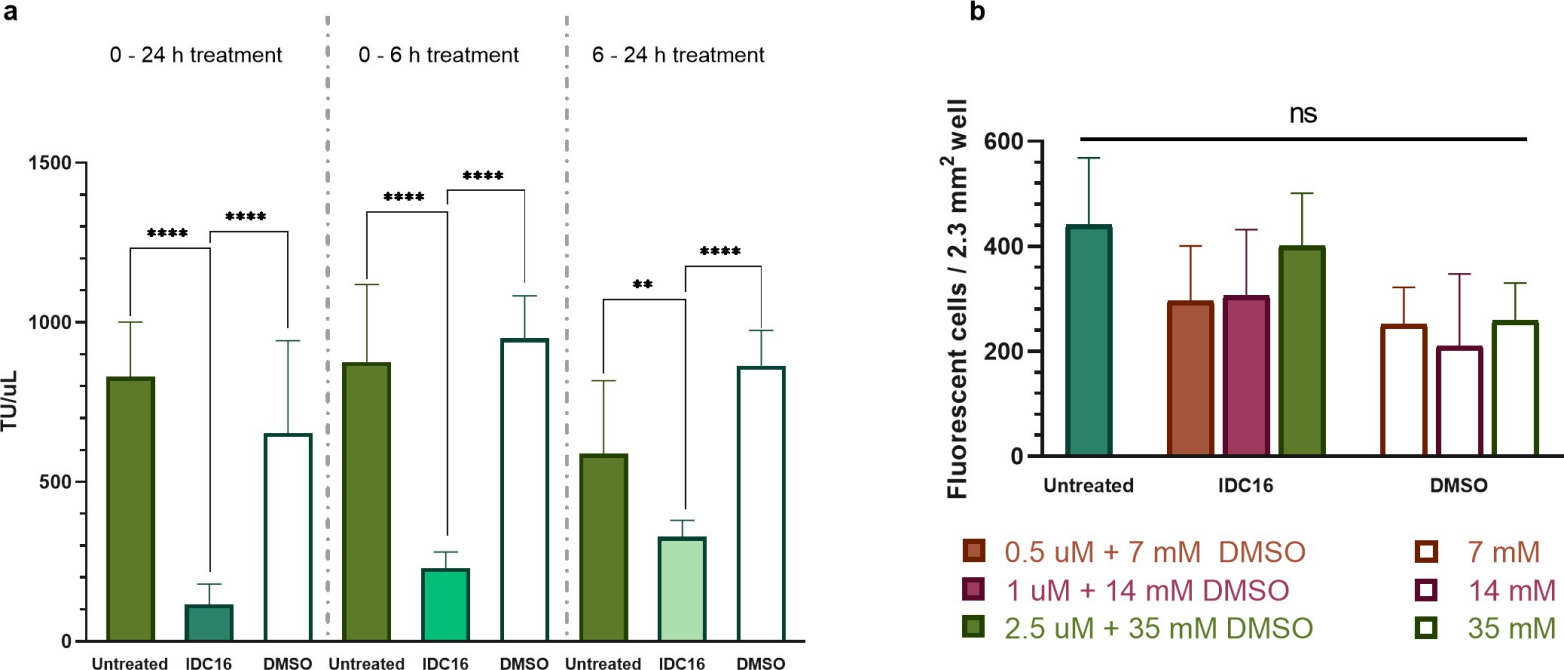
Effect of timing and duration of IDC16 treatment on infectious LV production, and GFP expression 6 h post-transfection. (a) Timing and duration of IDC16 treatment modified impact on infectious LV. Left: TU/uL after exposing cells from 0 to 24 h post-transfection to 2.5 uM IDC16 + 35 mM DMSO, 35 mM DMSO alone, or untreated control (n = 6). Middle: TU/ul after exposing cells from 0 to 6 h post-transfection to the same treatments (n = 5 to 6). Right: TU/ul after exposing cells from 6 to 24 h post-transfection to the same treatments (n = 5 to 6). (b) Number of fluorescing HEK293T cells per 2.33 mm^2^ of well was measured by automatic fluorescent cell count after treatment by IDC16 across different dilutions (n = 5 to 6). Bars and error bars show mean and SD. ** indicates significant differences at p ≤ 0.01, **** at p ≤ 0.0001, and. ns are ‘not significant’ differences.

Percentages of decrement by IDC16 in Figs 3–6 were calculated with $1-\frac{IDC16\;value}{untreated\;or\;DMSO\;value}$ as numerator and denominator. Other percentage calculations are specified in the text.

## Results

Lentiviral vectors were propagated in adherent HEK293T cells via transient, PEI-based transfection of confluent cells by backbone and packaging plasmids with or without treatments (none, small molecule + DMSO, or DMSO) at various concentrations. Six hours after transfection, overlay media containing treatments was removed and cells were washed. Fresh media was then added containing transfection enhancer plus treatment. Cells that were untreated or that were exposed to DMSO at a concentration equal to that of the small molecule treatment(s) served as controls. Twenty-four hours after transfection, supernatant and cell lysate were harvested. An aliquot of supernatant was transferred to confluent HEK293T cells in a new plate to measure infectious virus as transduction units (TUs). RNA was extracted from remaining supernatant and cell lysate in order to measure total and unspliced genome RNA viral copies in comparison with RNAseP housekeeping gene.

GFP expression and growth of transfected cells were monitored using fluorescence and brightfield microscopy, respectively. GFP expression increased from 6 to 24 h post-transfection in transfected cells (Fig 1A). No GFP was expressed by non-transfected cells from 6 to 24 h post-transfection. At 6 h, DMSO-free transfected cells (untreated) exhibited 442 green-fluorescent cells per 2.3 mm^2^ of well on average compared with 211 for DMSO-treated transfected cells. Both fluorescence counts were significantly higher than for non-transfected cells (negative control) (untreated vs neg. ctrl. p ≤ 0.0001, DMSO vs neg. ctrl. p = 0.0109). The number of fluorescent cells for DMSO-free wells was also significantly higher than for DMSO-treated wells (p = 0.0056) (Fig 1C). Brightfield microscope images showed cell confluency increased post-transfection (Fig 1A).

Production of virus by transfected cells was evaluated by cell transduction assay to monitor infectious virions and by real-time quantitative polymerase chain reaction (RT-qPCR) to measure viral genome copies. Spent media cultured with transfected cells was pipetted from wells and centrifuged to yield cell-free supernatant. Supernatant harvested 24 h post-transfection contained 8x10^5^ to 1.1x10^6^ viral genome copies per uL in DMSO-free (untreated) and DMSO-treated transfected cells, respectively (Fig 1D). Both were significantly higher than in non-transfected cells (untreated vs. neg. ctrl. p ≤ 0.0001, DMSO vs. neg. ctrl. p ≤ 0.0001). Genome copies per uL in DMSO-free cells were also significantly higher than in DMSO-treated cells (p = 0.0326).

Supernatant was aliquoted to transduce fresh HEK293T cells in a cell transduction assay (Fig 1B). At 48 h post-transduction, this yielded 829 transducing units (TUs)/uL of supernatant from DMSO-free transfected cells and 391 TU/uL in DMSO-treated cells (Fig 1E). Both were significantly higher than in negative control cells (untreated vs. neg. ctrl. p ≤ 0.0001, DMSO vs. neg. ctrl. p < 0.001). TU/uL from DMSO-free cells was also significantly higher than DMSO-treated cells (p < 0.001).

The measured ratio of total viral genome copies to TUs was within ranges previously reported for lentiviral vector propagation [47, 85, 86]. Measured ratios of 1160 and 3564 for DMSO-free and DMSO-treated transfected cells, respectively, were within a range of 10^2^–10^4^ reported previously (Fig 1F).

Three independent cell transfections yielded consistent cell transduction assay and RT-qPCR results (Fig 2). Cell transduction assay of infectious LV was between ~600–800 TU/uL for the transfections (Fig 2A). Total and unspliced viral genome copies in extracts from these cell transfections were ~5.6x10^5^ to 7.6x10^5^ per uL (Fig 2B) and ~3.8x10^5^ to 5.2x10^5^ per uL (Fig 2C), respectively. DNA plasmid (pLJM1-EGFP Addgene #19319) was used to create standard curves for absolute quantification of the analyzed sequences. Fig A in S2 File shows standard curves for total and unspliced viral genomes across different LV productions with corresponding linear regressions (see S3 File for detailed regression analysis). Increased amplification efficiency of total genome viral copies relative to unspliced viral genome copies was attributed to differing primer pair affinities to the template.

Two small organic molecules reported to inhibit splicing of HIV-1 in cell culture [45, 46, 51] were evaluated to examine their effects on LV production and HEK293T cell growth using the transfection method described above. The indole derivative IDC16 exhibited significant modulation of LV productivity in the evaluation, whereas Filgotinib showed no effect (see S2B Fig in S2 File). Further study focused on the indole derivative.

Indole derivative IDC16 increased the relative number of unspliced genomes extracted in lysates from transfected cells. Fig 3A shows that adding IDC16 at 0.5 to 2.5 uM in culture media from 0 to 24 hours post-transfection increased the ratio of unspliced RNA genome copies to total RNA genome copies in cells. The increase was significant at 1 (p = 0.0377) and 2.5 uM (p < 0.001). At 0.5 uM IDC16, the ratio trended higher but was not statistically significant. The proportional increase in unspliced viral genomes was attributed to inhibition of lentiviral RNA genome splicing by IDC16, as reported previously in HIV-1-producing cells [45].

Splicing inhibitor IDC16 reduced production of infectious LV by HEK293T cells in a dose-dependent manner as shown in Fig 3B and 3C. Treatment with 1.0 to 2.5 uM IDC16 from 0 to 24 h post-transfection significantly decreased TU/uL of transfected supernatants compared to untreated cells (p = 0.0018 and p ≤ 0.0001, respectively) and DMSO-treated controls (p = 0.0018 and p ≤ 0.0001, respectively). The reduction in TU/uL increased significantly (p = 0.0043) from >56% at 1.0 uM to >70% at 2.5 uM IDC16. A lower dose, 0.5 uM, decreased TU/mL by 66% relative to untreated controls (p ≤ 0.0001), similar to 1 uM (p ≤ 0.0001). The highest dose, 2.5 uM, decreased TU/mL by >82% relative to untreated controls (p ≤ 0.0001), which was also significantly lower than DMSO-treated cells (p ≤ 0.0001).

IDC16 adversely affected cell proliferation and cell morphology at 10 uM in cultured adherent cells. Indoles like IDC16 comprised of planar aromatic rings are reported to intercalate DNA and/or inhibit topoisomerase II activity [87–89]. These effects could diminish cell proliferation and consequently LV production. Fig 4 shows that transfected cells counted in wells treated for 24 h post-transfection with 0.5, 1, and 2.5 uM IDC16 trended lower than DMSO-free transfected cells. Quantitative differences, however, remained insignificant (Fig 4A). In contrast, at 10 uM IDC16 in 140 mM DMSO (Fig 4B), both IDC16 in DMSO (p < 0.001) and DMSO alone (p = 0.0290) significantly reduced the number of cultured cells compared to untreated transfected cells. IDC16 at 10 uM also significantly decreased cell quantification compared to 140 mM DMSO control (p = 0.0179) and 2.5 uM IDC16 (p = 0.0005). Corresponding brightfield images (Fig 4C) illustrate the observed differences. The morphology of cells treated with 10 uM IDC16 in 140 mM DMSO was adversely affected beyond DMSO alone. Tight clusters of ragged cells observed at 10 uM IDC16 were not found in 140 mM DMSO alone. Confluency of cells treated with 140 mM DMSO alone remained lower than either untreated cells or cells exposed to 2.5 uM IDC16.

IDC16 damped an exponential LV production trajectory that was observed in transfected cells. Fig 5A compares infectious LV produced by HEK293T cells that had been transduced with supernatant collected at 10, 16, and 24 h post-transfection from IDC16-treated, untreated, and DMSO-only controls. At 10 h post-transfection, TU/uL values were comparable for IDC16- and un-treated cells. The TU/ul for DMSO control was lower than 2.5 uM IDC16 (p = 0.0336) and untreated control cells (p = 0.0061). At 16 and 24 h post-transfection, TU per uL from IDC16-treated cells decreased relative to untreated and DMSO control cells. Specifically, IDC16 lowered production of functional virus by 54% (p = 0.0279) at 16 h and 74% (p ≤ 0.0001) at 24 h compared to untreated cells. Compared to the DMSO control, IDC16 reduced 71% (p ≤ 0.0001) of the viral production at 16 h and 76% (p ≤ 0.0001) at 24 h. Relative to untreated cells, those treated with DMSO alone produced about 62% more LV at 16 h (p = 0.0166; (DMSO/untreated)-1), and no distinguishable difference at 24 h.

In Fig 5B, LV production in untreated cells, DMSO control, and IDC16 treatment were regressed to an exponential (see S4 File for detailed regression analysis). Untreated cells and DMSO controls produced significantly more functional LV after 24 h than IDC16, with corresponding doubling times of 2.452, 2.424, and 3.053 h, respectively. The coefficients of determination for each fit are shown beneath Fig 6B. Each R^2^ value was > 0.90.

Early and extended treatment of transfected cells with IDC16 decreased virus production the most. Transfected cells were exposed to IDC16 across different periods post-transfection to study effects at early and late periods of plasmid trafficking, virus production, and cell proliferation. Fig 6A compares treatment by 2.5 uM IDC16 in 35 mM DMSO from 0 to 24 h post-transfection (left), from 0 to 6 h post-transfection (center), and from 6 to 24 h post-transfection (right). Supernatants collected 24 h post-transfection were used to transduce HEK293T cells and quantitate cell transduction. Extended treatment from 0 to 24 h post-transfection decreased functional LV production most—86% and 82% (1 –IDC16/untreated or DMSO) relative to DMSO-free (p ≤ 0.0001) and DMSO-containing controls (p ≤ 0.0001), respectively. Early and reduced treatment from 0 to 6 h post-transfection decreased functional LV production less—74% and 76% (1 –IDC16/untreated or DMSO) relative to the DMSO-free (p ≤ 0.0001) and DMSO-containing controls (p ≤ 0.0001), respectively. Delayed and reduced treatment from 6 to 24 h post-transfection decreased functional LV production the least—44% and 62% (1 –IDC16/untreated or DMSO) relative to untreated cells (p = 0.0016) and DMSO control cells (p ≤ 0.0001). Delay and/or reduction of the treatment period increased but did not recover virus production.

GFP expression trended lower in cells treated with IDC16 from 0 to 6 h post-transfection at concentrations from 0.5 to 2.5 uM and in DMSO-treated controls. Fig 6B shows GFP expression at 6 h post-transfection was 442 cells per well (untreated), 297 cells per well (0.5 uM IDC16), 306 cells per well (1 uM IDC16), and 402 cells per well (2.5 uM IDC16). GFP expression for corresponding DMSO-only treated cells was 252 cells per well (7 mM DMSO), 211 cells per well (14 mM DMSO), and 259 cells per well (35 mM DMSO).

## Discussion

This study showed infectious LV production from HEK293T host cells in adherent culture in 96-well plates using PEI transfection. A 3:2 ratio of packaging to backbone plasmids was complexed with PEI in a 1:3 ratio and transfected at 0.57 ug DNA / 10^6^ cells to maximize LV yield [82, 90–93] and minimize cytotoxicity [94, 95]. PEI facilitates endocytotic uptake of DNA [96, 97] more economically than lipid transfection agents and with lower cytotoxicity and more stable LV yields than calcium phosphate, as it is less sensitive to pH [27, 98]. At 6 h post-transfection, transfecting media was replaced by OptiMEM containing KnockOut Serum Replacement (KSR) with sodium butyrate (NaBu) to enhance LV yield [52, 99–102]. KSR sustains cell growth and stabilizes LV better than serum [52, 103] which stimulates the mammalian complement system to degrade retroviruses [104, 105]. NaBu reopens chromatin to increase transcription by inhibiting histone deacetylase, thereby improving LV titer [106–108] despite putative epigenetic silencing of episomal LV genome [109] and autophagic degradation of HIV-1 viral polyproteins [110]. NaBu was added at 6 h rather than 16 h post- transfection [102, 111, 112] to coincide with initiation of synthesis of HIV-1-related proteins at 5 to 6 h post-transfection [113].

This study showed high throughput and reproducible LV production via adherent culture in 96-well plate enabled facile, quantitative analysis of IDC16 effects on alternative splicing [39, 40], LV yield, and cell proliferation within 24 h post-transfection. Sans modulator, LV yield was ~10^5^ TU/mL at 24 h post-transfection (Fig 2A), comparable to ~10^5^ TU/mL at 24 h post-transfection reported for suspension culture [82–84, 93, 114]. Effects of IDC16 on LV yield appeared within 24 h post-transfection. At 48 to 72 h post-transfection, reported LV yields rise to 10^6^ to 10^7^ TU/mL pre-concentration [25, 82, 92, 114, 115]. Titration of LV yield by cell transduction and fluorescence microscopy correlates with gene transfer efficiency [47], which is overestimated 102–10^4^-fold by RT-qPCR of backbone vector expression [47, 85, 86]. RT-qPCR, however, is faster and readily replicated [47], thereby supporting concomitant analysis of IDC16 effects on post-transcriptional splicing.

This study found that IDC16 altered splicing of LV backbone transcripts in PEI-transfected HEK293T cells. IDC16 at 1 and 2.5 uM increased unspliced LV genome copies relative to total copies in a dose-dependent manner at 24 h post-transfection (Fig 3A). In human osteosarcoma (HOS)-CD4+-CCR5+ cells, Bakkour *et al*. showed IDC16 at 0.1 and 1 uM reduced multiply spliced HIV-1 species without changing unspliced species at 48 h post-infection [45]. In HeLa cells, Soret *et al*. reported that IDC16 at 5 uM reduced spliced HIV-1 products at 48 h post-infection with HIV-1 [46]. Likewise, in HeLa cells, Shkreta *et al*. determined that IDC16 analog 1C8 at 1 uM reduced spliced HIV-1 variants treated for 24 h post-infection by HIV-1 [116]. Inhibition of splicing in transfected, 3^rd^-generation LV backbone vector in HEK293T hosts by IDC16 mirrors its activity on integrated HIV-1 genome despite marked truncation and segregation of the wild-type (wt) genome within engineered plasmids.

This study found that IDC16 suppressed HEK293T production of infectious LV by > 56% and 70% at 1 and 2.5 uM, respectively, after 24 h exposure (Fig 3B and 3C). In infected macrophages, Bakkour *et al*. reported dose-dependent decreases in HIV-1 production of 63% and 98% at 0.5 and 1 uM IDC16, respectively, after 7 days [45]. In *Mus dunni* tail fibroblasts, Keriel *et al*. showed IDC16 at 1 uM reduced retroviral murine leukemia virus (MLV) by 35% after 48 h [117]. In infected peripheral blood mononuclear cells (PBMCs), Shkreta *et al*. reported that IDC16 structural analog 1C8 suppressed HIV-1 multiplication by 60% and 80% at 1.25 and 2.5 uM, respectively [116]. Likewise, in infected PBMCs, IDC16 structural analog ABX464, inhibited HIV-1 replication by 65% and 90% after treatment for 6 days with 1.1 and 3.3 uM, respectively [118]. The present study directly showed IDC16 reduced infectious LV using cell transduction, whereas all prior studies of IDC16 and its analogs on HIV-1 and MLV inferred viral replication was inhibited by titration of capsid protein p24 [45, 116] or envelope viral protein H48 [117], respectively. Nonetheless, reduced replication of HIV-1 or MLV by IDC16 or its analogs was attributed to altered viral splicing [45, 116, 118].

Unlike HIV-1 replication which depends on post-transcriptional splicing [39, 40], production of LV via the 3^rd^ generation system is designed to be independent of splicing [119]. Wt HIV-1 transcripts are spliced to yield gene products essential to transcribe, transport, translate, assemble, and bud infectious virus [39, 40], whereas 3^rd^ generation LV backbone and three accessory plasmids yield a minimal set of unspliced gene products that enable transport, translation, assembly, and budding of functional LV [119]. Consequently, prior work to condense LV backbone plasmid reported that abrogation of unintended splicing of LV backbone transcripts could expand the reservoir of packageable LV genomes thereby enhancing LV productivity [38].

This study found that, despite increasing unspliced transcripts in HEK293T cells transfected by 3^rd^ generation plasmids, IDC16 decreased yield of functional LV in a dose-dependent manner. This indicated that effects of IDC16 beyond reduced splicing of transgene transcripts contributed to the sharp decline in vector productivity. So, effects of IDC16 treatment on cell proliferation, the time-course of viral production, and influences of timing and duration of IDC16 treatment on LV productivity were examined.

High concentrations of IDC16 adversely affected cell proliferation. The number of adherent transfected HEK293T cells decreased by ~62% and 35% after 24 h post-transfection when treated with 10 uM IDC16 compared to untreated and DMSO-treated cells, respectively (Figs 3C and 4B). Similarly, Cheung *et al*. observed a 70–80% cell viability drop in CEM-GXR cells exposed to 8 and 16 uM IDC16 for 24 h, respectively [120]. Harmenberg *et al*. reported that indole derivative B-220 decreased proliferation of human embryonic rhabdomyosarcoma cells by 27% and 65% after treatment with 10 and 100 uM, respectively [121]. Likewise, Poljaková *et al*. reported a nearly complete loss of cell viability in 4 different neuroblastoma cell lines exposed to 10 uM ellipticine, a molecule that is structurally similar to IDC16 [87]. The reported cytotoxicity of indole derivatives and analogs has been attributed to intercalation of the planar ring structure of the small molecule into double-stranded (ds)DNA and/or inhibition of topoisomerase II activity [87–89, 122].

Lower concentrations of IDC16 showed a less observable effect on cell proliferation than higher concentrations. IDC16 at 0.5 and 2.5 uM did not significantly affect HEK293T cell count at 24 h post-transfection (Fig 4A). Similarly, Bakkour *et al*. established no adverse effect on cell proliferation in HIV-1 infected PBMCs up to 2.5 uM IDC16 [31]. Cheung *et al*., however, determined that in CEM-GXR cells the observable cytotoxicity threshold for IDC16 was 1 uM [120]. A ~10% drop in cell viability from 1 to 2 uM IDC16 was observed, prefacing an expectation of cytotoxicity from long-term exposure to minimal IDC16 concentrations [120]. So, we analyzed the time-course of exposure to IDC16 on LV productivity.

This study found that lower concentrations of IDC16 impaired LV production as the period of exposure increased. At 10 h post-transfection there was no observable difference in infectious LVs produced in treated cells (Fig 5A). At 16 and 24 h post-transfection, IDC16 reduced production of functional virus by 54% and 74%, respectively, compared to untreated cells. The time-course of LV production in untreated cells, DMSO controls, and IDC16-treated cells fit well to an exponential (Fig 5B), with corresponding doubling times of 2.452, 2.424, and 3.053 h, respectively. In an exponential production trajectory, 10 hours post-transfection was too soon to distinguish differences in LV yield in treated vs. untreated and control cells. IDC16 effect on yield became apparent at 16 hours in our adherent format. By comparison, suspended cells in a perfusion system were reported to begin budding LVs at around 20 h post-transfection [123]. In our adherent format, introducing production enhancement media at 6 h post-transfection washed out some transfected cells, and/or slowed cell growth, which both would diminish vector productivity at 10 h post-transfection and suppress observable differences. DMSO may have contributed to higher cell washout [124].

Overall, IDC16 diminished LV production with an exponential trajectory during 24 h post-transfection. This decrease could arise from IDC16 effects on the HEK293T cell cycle, retro-transduction of HEK cells by recently produced vesicular stomatitis virus glycoprotein (VSV-G) pseudotyped LVs, and PEI-based gene delivery. The exponential time-course of LV production mirrors cell count in log growth. Mitotic disintegration of the nuclear membrane is a primary mechanism for heterologous gene delivery to the nucleus [125–128]. Daughter cells then express inherited plasmids, producing LVs. Reported intercalation of IDC16 into double-stranded (ds)DNA and/or inhibition of topoisomerase II activity [87–89, 122] would disrupt mitosis, slowing nuclear uptake of transfected plasmid.

LV yield at 24 h post-transfection resulted from production by co-transfected cells in addition to production via retro-transduction of producer cells. VSV-G pseudotyped LVs can transduce a broad range of cell types including HEK cells [129, 130]. Producer cells are thence susceptible to retro-transduction—infection by newly budded LV—which increases viral titer [131, 132] even in a self-inactivating third-generation VSV-G pseudotyped LV [133–135]. Ohishi *et al*. showed that retro-transduction increased with a rise in VSV-G pseudotyped HIV-1 production by calcium-phosphate transfected HEK293T cells [131]. Diminishment of LV production by IDC16 due to mitotic disruption would delay the rise of LV titer, thereby reducing contribution of retro-transduction to LV titer [131, 132].

dsDNA intercalation is reported to disrupt transfection. El-Mogy and Haj-Ahmad reported that dsDNA intercalator ethidium bromide (EtBr) decreased the fraction of LacZ expressing Chinese hamster ovary (CHO) cells at 21 h after transfection with calcium phosphate at EtBr concentrations between 2.5–250 nM [136]. This was attributed to intracellular distortion of plasmid function and structure, rather than reduced plasmid uptake into cells, since a reduction in fluorescence of DNA intercalated by EtBr was observed in the presence of cationic polymers and lipids that were reported to displace EtBr by complexation with the DNA [137–140]. dsDNA intercalation by IDC16 [87–89, 122] could similarly disrupt post-uptake plasmid trafficking, countering PEI activity, and thereby plasmid expression. PEI enables transfection in a series of ways from uptake of plasmid to its nuclear import. PEI facilitates foreign DNA cell uptake [97, 141–143], endosomal escape [96, 125, 144–146], the cytoplasmatic journey to nuclear space [147–149], and the import of DNA to the nucleus [126, 150] for transgene expression and subsequent protein translation and LV assembly.

IDC16 disruption of intracellular plasmid trafficking, cell cycle, and retro-transduction would each contribute to reduced LV production. Disruption to plasmid trafficking would primarily occur earlier in transfection, ultimately lowering LV production via transiently transfected and retro-transduced producer cells. So, treatment of transfected cells by IDC16 at earlier and later periods of transfection was compared to ascertain the potential import of plasmid trafficking disruption on reduced LV production. Effects of timing and duration of indole-related exposure on viral production have not previously been reported.

This study found that IDC treatment at transfection affected LV yield more than later treatment. IDC16 treatment from 0 to 6 h post-transfection reduced LV yield more than treatment from 6 to 24 h post-transfection (Fig 6A). Removal of IDC16 at 6 h post-transfection coincided with the previously reported onset of HIV-1-related protein production at 5 or 6 h post-transfection [113]. Production of functional LV was partially recovered by removing IDC16 at 6 h (Fig 6A). Limiting IDC16 treatment to between 6 and 24 h recovered more production but did not restore it. Moreover, GFP-expressing HEK293T cells at 6 h post-transfection trended lower than the untreated control, although differences remained insignificant (Fig 6B). Together, these data suggested disruption of post-uptake plasmid trafficking by IDC16 intercalation of dsDNA may be significant to its reduction of LV titer. Such an effect, occurring earlier in transfection, outweighed effect of IDC16 on e.g., subsequent post-transcriptional processing, assembly, or budding of LV. Downward, but not significant, trends in cell proliferation at 2.5 uM (Fig 4A) suggested IDC16 effects on cell cycle may also contribute to reduced LV titer.

Reported inhibition by IDC16 of the RNA-binding protein SF2/ASF may decrease LV titer in ways besides post-transcriptional processing. In addition to regulation of splicing, SF2/ASF is involved in the control of transcription activation, messenger (m)RNA translation, and mRNA nuclear export [151]. Ji *et al*., overexpressed SF2/ASF in transfected HEK293T cells and showed HIV-1-long terminal repeat (LTR) promoter was activated, increasing luciferase mRNA, reflecting enhanced transcription and transduction [152]. Yet overexpression of SF2/ASF in LV producer cells may result in different outcomes due to the changes in HIV-1-LTR promoters in 3^rd^-generation LVs. Moreover, IDC16 may also alter mRNA nuclear export because it is associated with the phosphorylated state of SF2/ASF [153] similar to splicing [154].

## Conclusion

Overall, indole derivative IDC16 increased the relative level of unspliced viral genome in transfected cells. The increase coincided with diminished production of functional virus from transient transfection of adherent HEK293T cells in a dose-dependent manner. Adjusting the period of IDC16 treatment suggested that IDC16 disruption of early stages of transfection and cell cycle attenuated the exponential trajectory of virus production more than its alteration of splicing. Diminution of viral production by IDC16 was curtailed for shorter and/or delayed treatment post-transfection. Proliferation and morphology of transfected HEK293T cells were significantly altered by 10 uM IDC16 treatment. GFP production by IDC16-treated cells at 6 h post-transfection was not significantly reduced, though it ostensibly trended lower. Relative influences of early-stage transfection, cell proliferation, retrotransduction, and post-transcriptional splicing on transient transfection of HEK293T cells for LV production are indicated by these results.

## Supporting information

S1 File Summary of statistical analysis. (PDF)

S2 File Supplementary figures. (DOCX)

S3 File Regression analysis of standard curves for total and unspliced viral genomes. (PDF)

S4 File Regression analysis of production trajectory of LV in TU/uL at 10, 16, and 24 h. (PDF)

S5 File Data. (XLSX)
