# Supplementary material for: Effects of an indole derivative on cell proliferation, transfection, and alternative splicing in production of lentiviral vectors by transient co-transfection
Source: PLoS One. 2024 Jun 4;19(6):e0297817. doi: 10.1371/journal.pone.0297817 (PMC11149887; doi:10.1371/journal.pone.0297817)
Supplement: S1 File — (PDF) [file pone.0297817.s001.pdf]

Fig 1c.
N = 6 in each group.
No outliers were identified by Tukey’s Hinges test.
One-way ANOVA assumptions

Independence of observations: passed. Data was collected from different wells in each group.

Normality: Neg. ctrl did not pass. Other groups passed.

Homogeneity of variance: passed.

Transformation of data did not fulfill the normality test in neg. ctrl. However, the test was applied because 4 values are zero and the others are 2 and 3. This was because the negative control had, as expected, little or no fluorescent cells. Most of the transformations resulted in the loss of values without any improvement in normality.

Fig 1d.
N = 6 in each group.
One-way ANOVA assumptions

Independence of observations: passed. Data was collected from different wells in each group.

Normality: passed.

Homogeneity of variance: passed.

Complete data

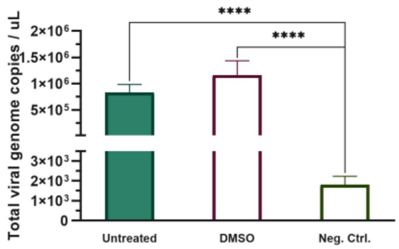

Two outliers were identified by Tukey’s Hinges test.
N = 6 for DMSO and N = 5 for untreated and neg. ctrl
One-way ANOVA assumptions

Independence of observations: passed. Data was collected from different wells in each group.

Normality: passed.

Homogeneity of variance: passed.

Eliminating outliers adds a significant difference between untreated and DMSO.

Excluding outliers

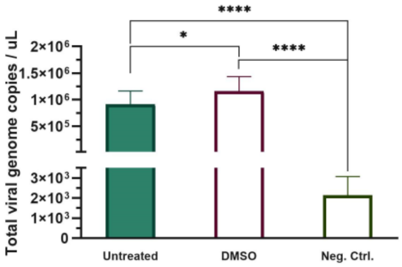

Fig 1e.
N = 6 in each group.
No outliers were identified by Tukey's Hinges test.
One-way ANOVA assumptions

Independence of observations: passed. Data was collected from different wells in each group.
Normality: passed.
Homogeneity of variance: passed.

Fig 1f.
No statistical analyses were performed. The ratio in negative control gives an undefined result (division by zero).

Fig 2a.
N = 6 for experiments 1 and 3. N = 4 for experiment 2. Experiment 2 was designed with only 4 repetitions.
No outliers were identified by Tukey's Hinges test.
One-way ANOVA assumptions

Independence of observations: passed. Data was collected from different wells in each group.
Normality: passed.
Homogeneity of variance: passed.

Fig 2b.
N = 6 for experiments 1 and 3. N = 4 for experiment 2. Experiment 2 was designed with only 4 repetitions.
One-way ANOVA assumptions

Independence of observations: passed. Data was collected from different wells in each group.
Normality: passed.
Homogeneity of variance: passed.

Complete data

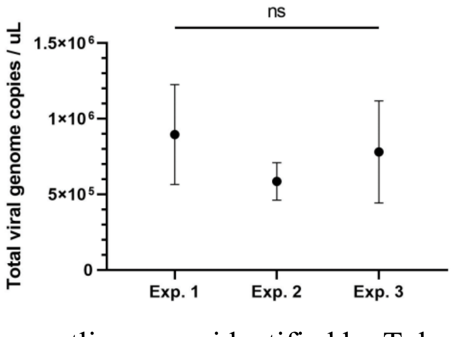

Two outliers were identified by Tukey's Hinges test.
N = 5 for experiments 1 and 3. N = 4 for experiment 2.
One-way ANOVA assumptions

Independence of observations: passed. Data was collected from different wells in each group.
Normality: passed.
Homogeneity of variance: passed.

Excluding the outliers changed the error bars but not the insignificance of the results.

Excluding outliers

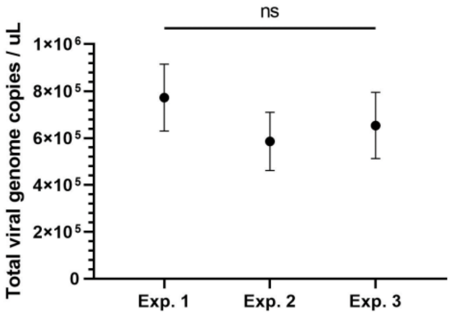

Fig 2c.

N = 6 for experiments 1 and 3. N = 4 for experiment 2. Experiment 2 was designed with only 4 repetitions.

One-way ANOVA assumptions

Independence of observations: passed. Data was collected from different wells in each group.
Normality: Experiment 3 did not pass. Other groups passed.
Homogeneity of variance: passed.

Data was transformed to reciprocal to fulfill all ANOVA assumptions. Transforming the data did not change the insignificance of the results.

Complete transformed data

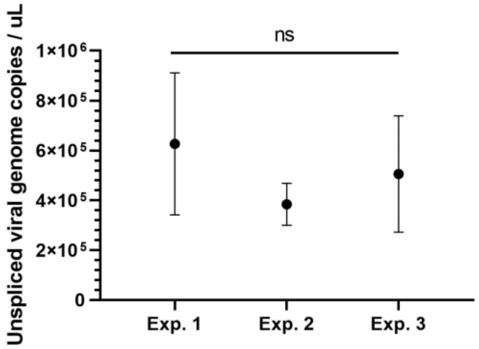

Two outliers were identified by Tukey's Hinges test.

N = 5 for experiments 1 and 3. N = 4 for experiment 2.

One-way ANOVA assumptions

Independence of observations: passed. Data was collected from different wells in each group.
Normality: passed.

Homogeneity of variance: passed.

Excluding the outliers changed the error bars but not the insignificance of the results Also, excluding the outliers eliminated the need to transform the data to fulfill assumptions.

Excluding outliers

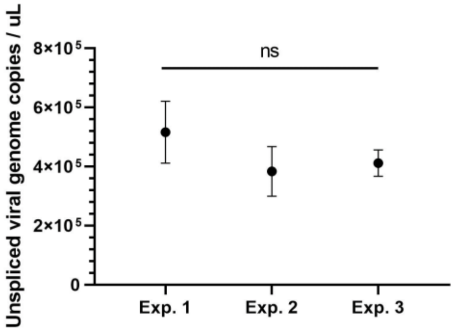

Fig 3a.

N = 6 in each group

No outliers were identified by Tukey's Hinges test.

One-way ANOVA assumptions

Independence of observations: passed. Data was collected from different wells in each group.

Normality: passed.

Homogeneity of variance: passed.

Fig 3b.

N = 4 in each group.

No outliers were identified by Tuckey's Hinges test.

One-way ANOVA assumptions

Independence of observations: passed. Data was collected from different wells in each group.

Normality: IDC16 1 uM group did not pass. Other groups passed.

Homogeneity of variance: not passed.

Data was transformed to reciprocal to fulfill all ANOVA assumptions. Transforming the data and fulfilling assumptions created a significant difference between IDC16 1 uM and IDC16 2.5 uM. Also, the significance of the differences increased.

Complete data Complete transformed data

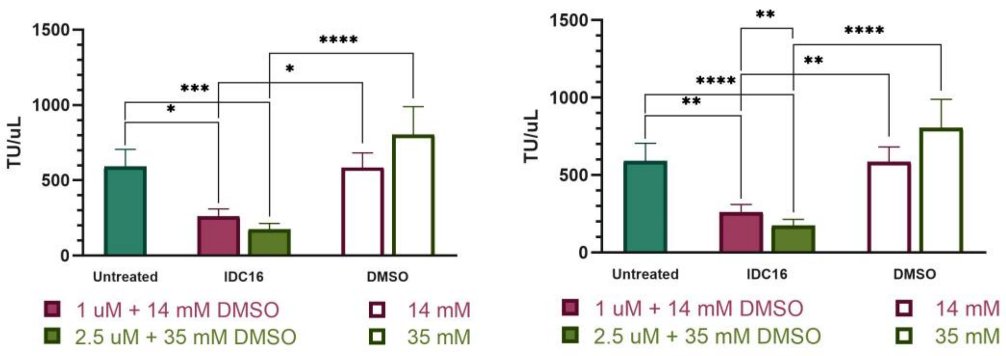

Fig 3c.
N = 6 in each group
No outliers were identified by Tuckey's Hinges test.
One-way ANOVA assumptions

> Independence of observations: passed. Data was collected from different wells in each group.
> Normality: passed.
> Homogeneity of variance: passed.

Fig 4a.
N = 3 in each group
No outliers were identified by Tuckey's Hinges test.
One-way ANOVA assumptions

> Independence of observations: passed. Data was collected from different wells in each group.
> Normality: passed.
> Homogeneity of variance: passed.

Fig 4b.
N = 4 in each group.
No outliers were identified by Tuckey's Hinges test.
One-way ANOVA assumptions

> Independence of observations: passed. Data was collected from different wells in each group.
> Normality: The DMSO 140 mM group did not pass. Other groups passed.
> Homogeneity of variance: not passed.

Data was transformed to reciprocal to fulfill all ANOVA assumptions.
Transforming the data and fulfilling assumptions created a significant difference between IDC16 10 uM and DMSO 140 uM. Also, the significance of the differences increased.

Complete data   Complete transformed data

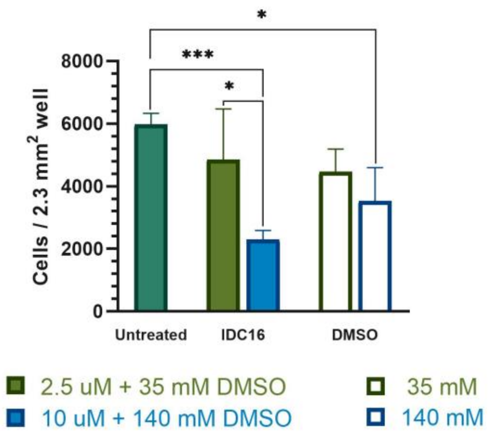

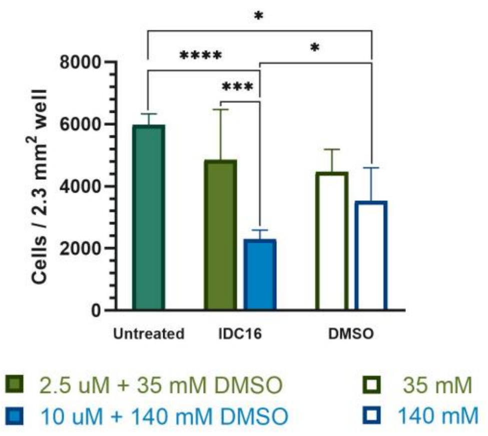

Fig 5a. panel 10 h

N = 6 for untreated and IDC16 2.5 uM. N = 5 for DMSO 35 mM. One sample in the DMSO group was lost in the experiment.

No outliers were identified by Tuckey's Hinges test.

One-way ANOVA assumptions

> Independence of observations: passed. Data was collected from different wells in each group.
> Normality: passed.
> Homogeneity of variance: passed.

Fig 5a. panel 16 h

N = 6 for IDC16 2.5 uM. N = 5 for IDC16 2.5 uM and DMSO 35 mM. A sample in each of those groups was lost in the experiment.

No outliers were identified by Tuckey's Hinges test.

One-way ANOVA assumptions

> Independence of observations: passed. Data was collected from different wells in each group.
> Normality: passed.
> Homogeneity of variance: passed.

Fig 5a. panel 24 h

N = 6 for untreated and DMSO 35 mM. N = 5 for IDC16 2.5 uM. One sample in the IDC16 group was lost in the experiment.

No outliers were identified by Tuckey's Hinges test.

One-way ANOVA assumptions

> Independence of observations: passed. Data was collected from different wells in each group.
> Normality: passed.
> Homogeneity of variance: passed.

Fig 6a. 0 – 24 h exposure
N = 6 in each group
No outliers were identified by Tuckey's Hinges test.
One-way ANOVA assumptions

> Independence of observations: passed. Data was collected from different wells in each group.
> Normality: passed.
> Homogeneity of variance: passed.

Fig 6a. 0 – 6 h exposure
N = 6 for untreated and DMSO 35 mM. N = 5 for IDC16 2.5 uM. One sample in the IDC16 group was lost in the experiment.
No outliers were identified by Tuckey's Hinges test.
One-way ANOVA assumptions

> Independence of observations: passed. Data was collected from different wells in each group.
> Normality: passed.
> Homogeneity of variance: passed.

Fig 6a. 6 – 24 h exposure
N = 6 for untreated and IDC16 2.5 uM. N = 5 for DMSO 35 mM. One sample in the DMSO group was lost in the experiment.
No outliers were identified by Tuckey's Hinges test.
One-way ANOVA assumptions

> Independence of observations: passed. Data was collected from different wells in each group.
> Normality: The untreated group did not pass. Other groups passed.
> Homogeneity of variance: passed.

Data was transformed to reciprocal to fulfill all ANOVA assumptions.
Transforming the data and fulfilling assumptions erased the significant difference between the untreated group vs DMSO 35 uM. Also, the significance of the differences increased.

Complete data

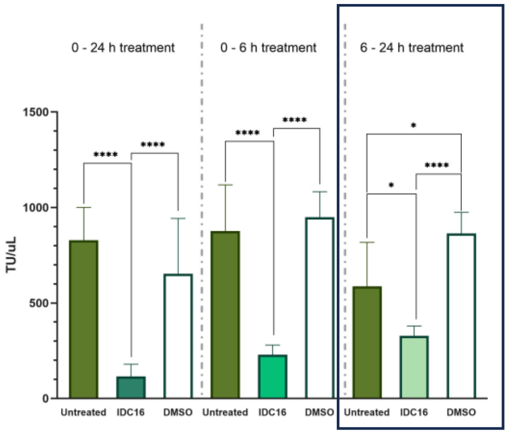

Complete transformed data

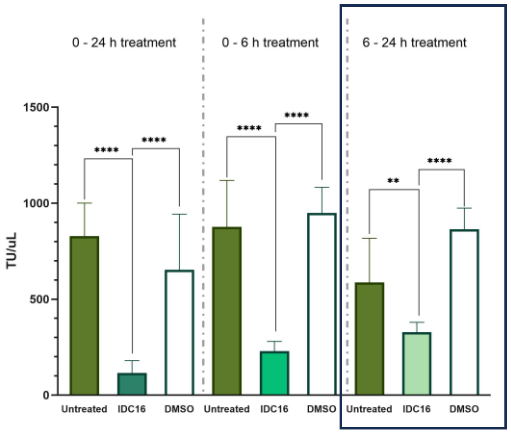

Fig 6b
N = 5 for DMSO 35mM. N = 6 in other groups. One sample in the DMSO group was lost in the experiment.
No outliers were identified by Tuckey's Hinges test.
One-way ANOVA assumptions

Independence of observations: passed. Data was collected from different wells in each group.

Normality: IDC16 2.5 uM group did not pass. Other groups passed.

Homogeneity of variance: passed.

Data was transformed to sin(X) to fulfill all ANOVA assumptions.
Transforming the data and fulfilling assumptions erased the significant difference between the untreated group and DMSO. Also, the significance of the differences increased.

Complete data

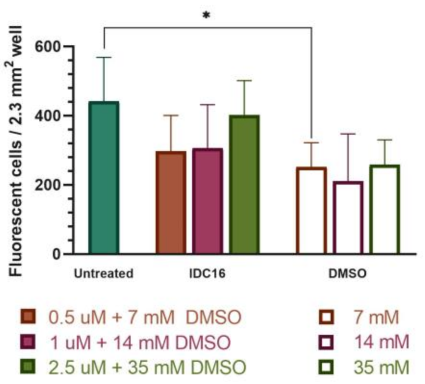

Complete transformed data

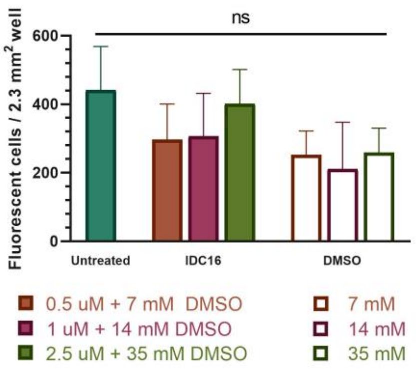

Fig Ba in S2 file.
N = 2 for Filgotinib 2.5 uM. N = 3 for DMSO 56 mM. N = 4 in other groups. Two samples in Filgotinib 2.5 uM and one sample in DMSO 56 mM were lost in the experiment.
No outliers were identified by Tukey's Hinges test.
One-way ANOVA assumptions

Independence of observations: passed. Data was collected from different wells in each group.

Normality: DMSO 140 mM did not pass. Other groups passed.

Homogeneity of variance: passed.

Transformation of data did not fulfill the normality test in DMSO 140 mM. Therefore, the Kruskal-Wallis test was applied for non-parametric data. No significant differences resulted in both ANOVA and the Kruskal-Wallis test.

Fig Bb in S2 file.
N = 4 in each group.
No outliers were identified by Tukey's Hinges test.
One-way ANOVA assumptions

Independence of observations: passed. Data was collected from different wells in each group.
Normality: passed.
Homogeneity of variance: passed.
