# Supplementary material for: Effects of an indole derivative on cell proliferation, transfection, and alternative splicing in production of lentiviral vectors by transient co-transfection
Source: PLoS One. 2024 Jun 4;19(6):e0297817. doi: 10.1371/journal.pone.0297817 (PMC11149887; doi:10.1371/journal.pone.0297817)
Supplement: S2 File — (DOCX) [file pone.0297817.s002.docx]

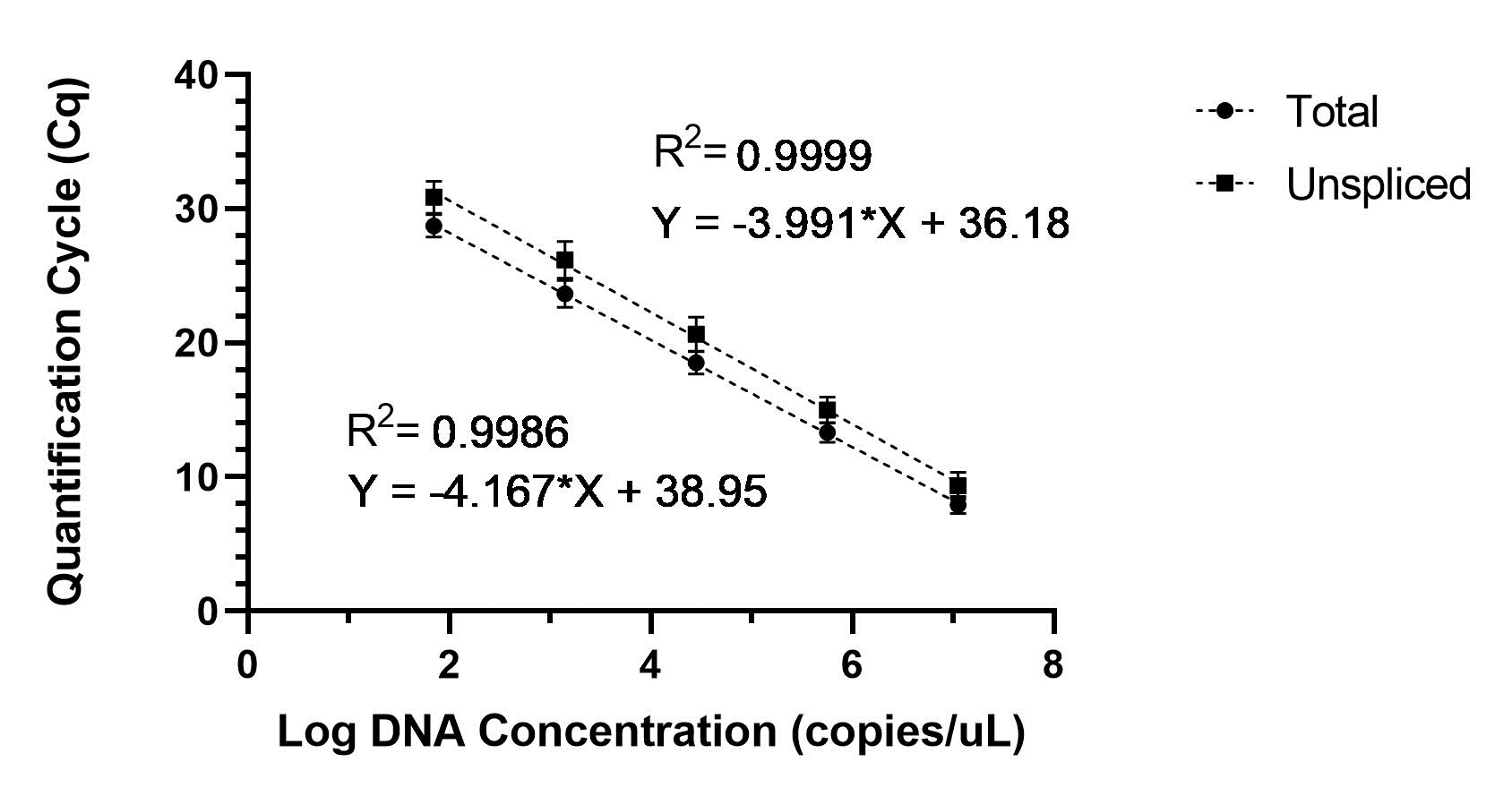


**Fig A. RT-qPCR** s**tandard curves.**

Average quantification cycles (Cq) of RT-qPCR calibration curves of backbone plasmid from 8 analyses of total and unspliced viral genomes.


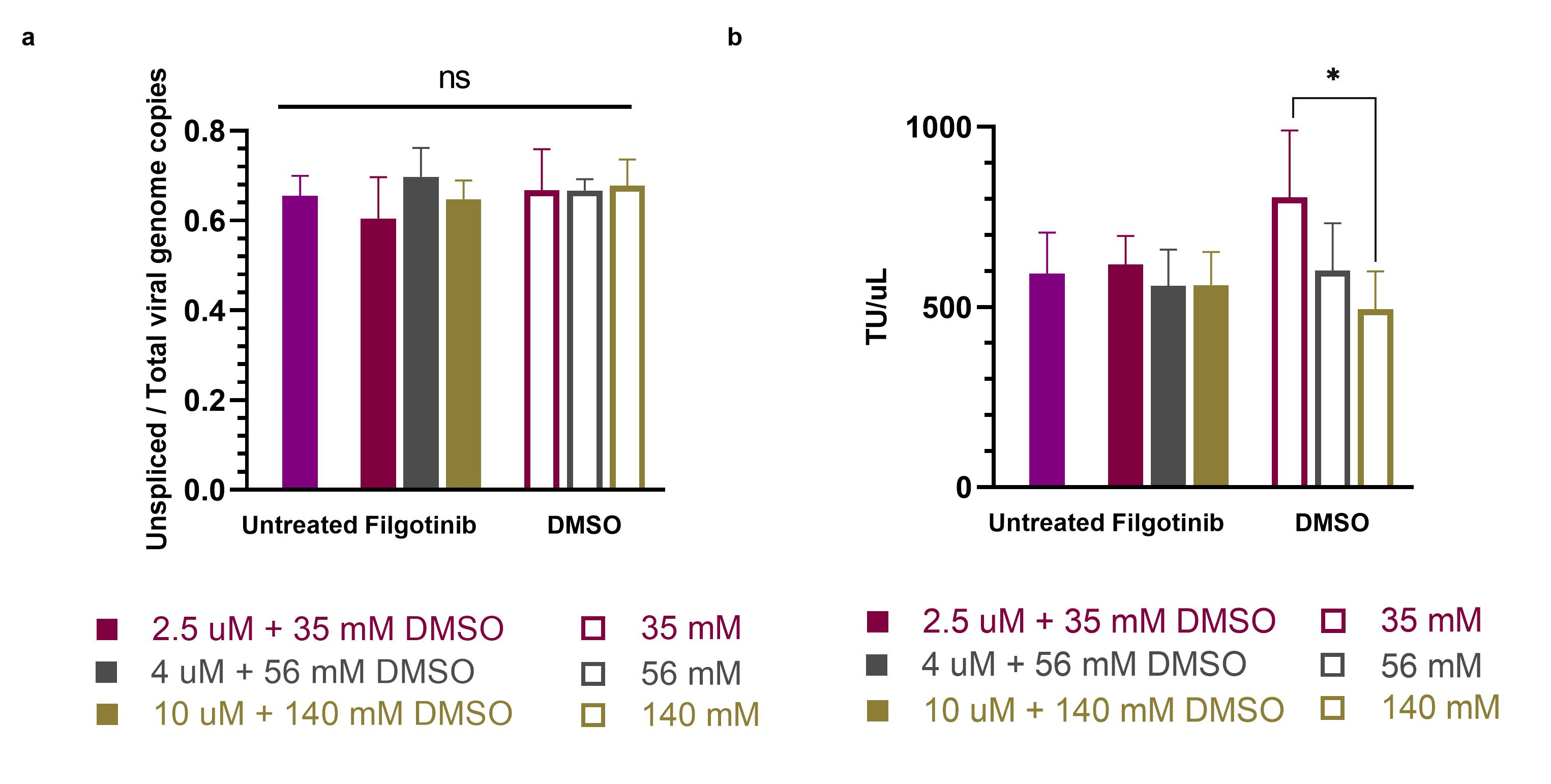


**Fig B.** **Filgotinib did not affect the proportion of unspliced viral genome copies or the production of infectious LVs.** (a) Ratio of unspliced RNA viral copies to total genome RNA viral copies measured by RTqPCR. (b) Infectious LV production (TU/uL) measured by cell transduction assay.

No significant differences were observed for 2.5 - 10 uM Filgotinib (solid magenta, grey, gold bars) dissolved in DMSO compared to DMSO-only controls (hollow magenta, grey, gold bars) or untreated controls (solid purple bars). Bars and error bars show mean and standard deviation (SD), respectively. * indicates significant differences at p ≤ 0.05 and ns are ‘not significant’ differences. Each point included 4 statistically valid replicates, except DMSO negative control at 56 mM (3 valid replicates) and 2.5 uM Filgotinib + 35 mM DMSO (2 valid replicates).
