# Supplementary material for: Effects of an indole derivative on cell proliferation, transfection, and alternative splicing in production of lentiviral vectors by transient co-transfection
Source: PLoS One. 2024 Jun 4;19(6):e0297817. doi: 10.1371/journal.pone.0297817 (PMC11149887; doi:10.1371/journal.pone.0297817)
Supplement: S3 File — (PDF) [file pone.0297817.s003.pdf]

Linear reg. Tabular results		A Total	B Unspliced
1	**Best-fit values**		
2	Slope	-3.991	-4.167
3	Y-intercept	36.18	38.95
4	X-intercept	9.066	9.346
5	1/slope	-0.2506	-0.2400
6			
7	**Std. Error**		
8	Slope	0.02625	0.08938
9	Y-intercept	0.1264	0.4305
10			
11	**95% Confidence Intervals**		
12	Slope	-4.075 to -3.907	-4.452 to -3.883
13	Y-intercept	35.78 to 36.58	37.58 to 40.32
14	X-intercept	8.964 to 9.172	9.010 to 9.727
15			
16	**Goodness of Fit**		
17	R square	0.9999	0.9986
18	Sy.x	0.1080	0.3677
19			
20	**Is slope significantly non-zer**		
21	F	23108	2174
22	DFn, DFd	1, 3	1, 3
23	P value	<0.0001	<0.0001
24	Deviation from zero?	Significant	Significant
25			
26	**Equation**	Y = -3.991*X + 36.18	Y = -4.167*X + 38.95
27			
28	**Data**		
29	Number of X values	5	5
30	Maximum number of Y replica	1	1
31	Total number of values	5	5
32	Number of missing values	0	0

Are the slopes equal?
F = 3.589. DFn = 1, DFd = 6
P=0.1070

If the overall slopes were identical, there is a 10.7% chance of randomly choosing
data points with slopes this different. You can conclude that the differences between the slopes are not significant.

Since the slopes are not significantly different, it is possible to calculate one slope for all the data.
The pooled slope equals -4.079.

Are the elevations or intercepts equal?
F = 97.55. DFn = 1, DFd = 7
P<0.0001

If the overall elevations were identical, there is a less than 0.01% chance of randomly
choosing data points with elevations this different. You can conclude that the differences between the elevations are extremely significant.
