# Supplementary material for: Effects of an indole derivative on cell proliferation, transfection, and alternative splicing in production of lentiviral vectors by transient co-transfection
Source: PLoS One. 2024 Jun 4;19(6):e0297817. doi: 10.1371/journal.pone.0297817 (PMC11149887; doi:10.1371/journal.pone.0297817)
Supplement: S4 File — (PDF) [file pone.0297817.s004.pdf]

Nonlin fit<br>Table of results		A<br>Untreated	B<br>2.5 uM IDC16 + 35 mM DMSO	C<br>35 mM DMSO
1	**Exponential growth equation**			
2	**Best-fit values**			
3	Y0	= 1.000	= 1.000	= 1.000
4	k	0.2824	0.2270	0.2860
5	Tau	3.541	4.405	3.497
6	Doubling Time	2.454	3.053	2.424
7	**95% CI (profile likelihood)**			
8	k	0.2773 to 0.2870	0.2220 to 0.2315	0.2825 to 0.2892
9	Tau	3.484 to 3.607	4.319 to 4.505	3.458 to 3.540
10	Doubling Time	2.415 to 2.500	2.994 to 3.122	2.397 to 2.454
11	**Goodness of Fit**			
12	Degrees of Freedom	22	22	21
13	R squared	0.9060	0.9049	0.9526
14	Sum of Squares	323178	18228	171370
15	Sy.x	121.2	28.78	90.34
16	**Constraints**			
17	Y0	Y0 = 1	Y0 = 1	Y0 = 1
18				
19	**Number of points**			
20	# of X values	60	66	72
21	# Y values analyzed	23	23	22

**Nonlin fit**<br>Summary table		A		B		C		D	
		Y0		k		Tau		Doubling Time	
		Value	SEM	Value	SEM	Value	SEM	Value	SEM
1	Untreated	1.000		0.282	0.002	3.541		2.454	
2	2.5 uM IDC16 + 35 mM DMSO	1.000		0.227	0.002	4.405		3.053	
3	35 mM DMSO	1.000		0.286	0.002	3.497		2.424	
